# Artificial neural networks versus LASSO regression for the prediction of long-term survival after surgery for invasive IPMN of the pancreas

**DOI:** 10.1371/journal.pone.0249206

**Published:** 2021-03-25

**Authors:** Linus Aronsson, Roland Andersson, Daniel Ansari

**Affiliations:** Department of Surgery, Clinical Sciences Lund, Lund University, Skåne University Hospital, Lund, Sweden; Wroclaw University of Science and Technology, POLAND

## Abstract

Prediction of long-term survival in patients with invasive intraductal papillary mucinous neoplasm (IPMN) of the pancreas may aid in patient assessment, risk stratification and personalization of treatment. This study aimed to investigate the predictive ability of artificial neural networks (ANN) and LASSO regression in terms of 5-year disease-specific survival. ANN work in a non-linear fashion, having a potential advantage in analysis of variables with complex correlations compared to regression models. LASSO is a type of regression analysis facilitating variable selection and regularization. A total of 440 patients undergoing surgical treatment for invasive IPMN of the pancreas registered in the Surveillance, Epidemiology and End Results (SEER) database between 2004 and 2016 were analyzed. The dataset was prior to analysis randomly split into a modelling and test set (7:3). The accuracy, precision and F1 score for predicting mortality were 0.82, 0.83 and 0.89, respectively for ANN with variable selection compared to 0.79, 0.85 and 0.87, respectively for the LASSO-model. ANN using all variables showed similar accuracy, precision and F1 score of 0.81, 0.85 and 0.88, respectively compared to a logistic regression analysis. McNemar´s test showed no statistical difference between the models. The models showed high and similar performance with regard to accuracy and precision for predicting 5-year survival status.

## Introduction

Intraductal papillary mucinous neoplasm (IPMN) of the pancreas is a cystic lesion arising from the ductal cells lining the pancreatic ducts. IPMN harbors a potential to become malignant (invasive). The likelihood of malignant transformation into invasive IPMN seems to in part be dependent upon the location of the lesion, i.e. main duct, branch duct or mixed type, as well as the histological differentiation, i.e. gastric, pancreatobiliary or intestinal [1,2].

The main treatment of invasive IPMN, as for other types of pancreatic cancer, is surgical resection. The role of neoadjuvant therapy is to date not fully established by the guidelines. Adjuvant chemotherapy, however, has recently been recommended [2,3] and shows benefit in selected cases, either as solely administered or in combination with radiotherapy [4]. Compared to pancreatic ductal adenocarcinoma (PDAC), invasive IPMN seems to have a superior postoperative prognosis [5]. This may be explained by earlier detection as well as biological differences. Invasive IPMN can be divided into different histological subtypes, the two most common being colloid and tubular, where the colloid subtype has improved survival compared to the tubular subtype [1,5].

Prediction models of postoperative survival is important for risk stratification and prognosis, which can improve further treatment as well as future planning. Five-year survival is used as a benchmark in cancer prognosis and is often reported as “cure” if no recurrence has occurred. Disease-specific survival captures death from residual or recurrent disease. Five-year disease-specific survival (DSS) is thus an important measure in cancer research.

Prediction models are traditionally based on regression analyses. The Least Absolute Shrinkage and Selection Operator (LASSO) [6] is a regression method especially valuable in datasets with numerous variables and support unbiased parameter selection. Variable selection is important for generalization, i.e. avoiding overfitting of dataset. In contrast to regression models, artificial neural networks (ANN) work in a non-linear fashion resembling biological neural networks [7]. This approach may have an advantage when predicting survival in cancer because of the ability to find underlying patterns in complex data. ANN have been used previously in pancreatic cancer [8] and have been shown to outcompete the prediction of the AJCC TNM-stage [9]. The major disadvantage of ANN revolves around its “black box” nature, which limits conclusions regarding relationship between variables as well as an increased risk of overfitting the prediction model [7].

The primary aim of this study was to create predictive models for 5-year DSS after surgery for invasive IPMN. ANN models (with and without selection of variables), LASSO models and logistic regression models were created. The performance of the models was investigated.

## Methods

### Data source

The Surveillance, Epidemiology and End Results (SEER) database (2004–2016) was queried. SEER is a prospective database, maintained by the National Cancer Institute (NCI). Data were obtained from all US cancer registries participating in the SEER program. Patients were identified on the basis of the International Classification of Diseases for Oncology, 3rd edition (ICD-O-3) for tumors of the exocrine pancreas: C25.0, C25.1, C25.2, C25.3, C25.7, C25.8, and C25.9. Cases with histopathologically confirmed invasive IPMN of the pancreas (ICD-O-3 histology codes 8050, 8260, 8450, 8453, 8471, 8480, 8481 and 8503 [10,11] were included. Patients that had not undergone surgical resection were excluded. Only adult patients (≥18 years old at diagnosis) were included. A total number of 912 eligible patients were identified. The Ethics Committee for Clinical Research at Lund University, Sweden, approved the study protocol (2016/100). The study followed the STROBE guidelines [12].

### Variables and baseline data

**Fig 1:**
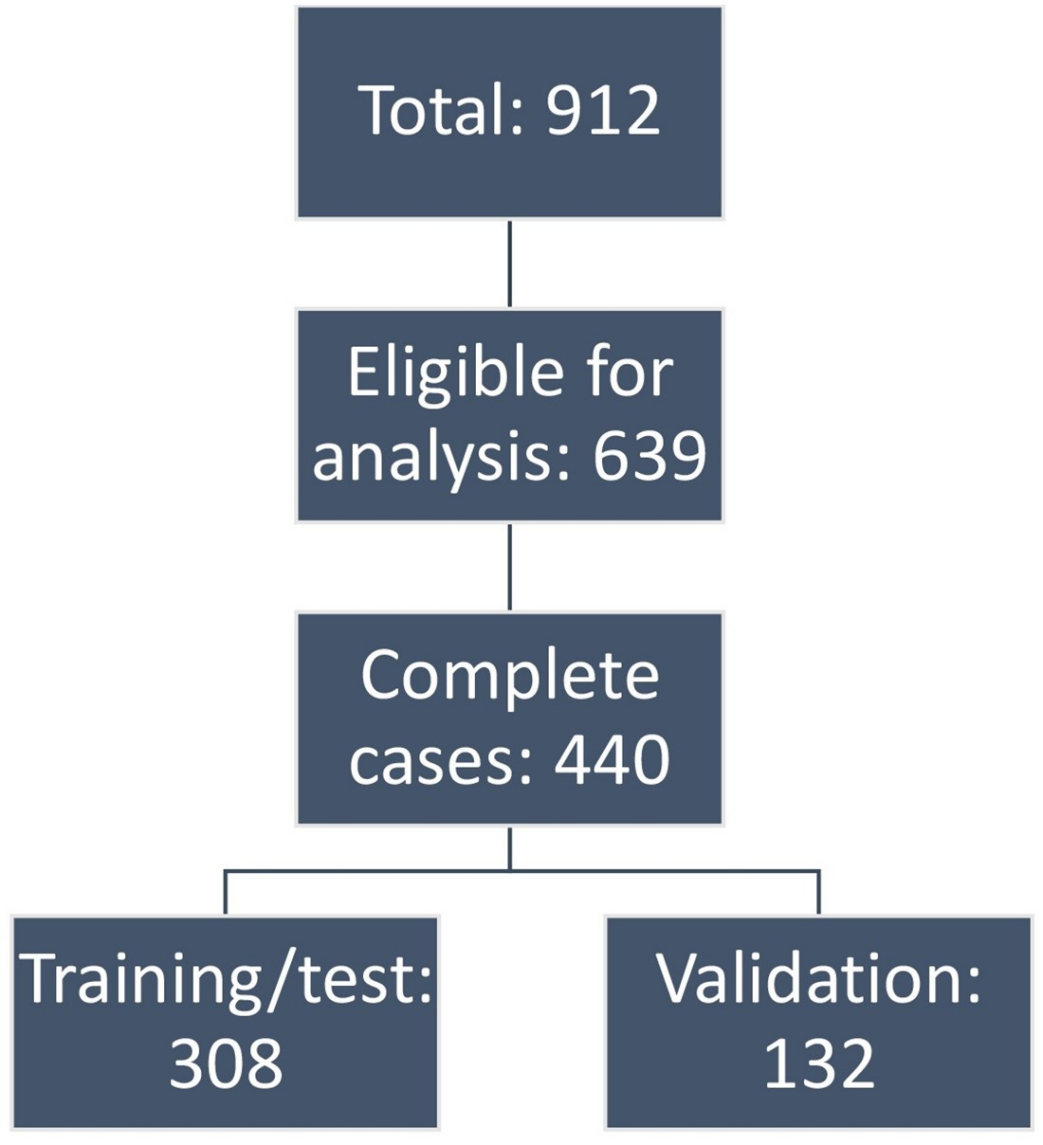
Flow chart of selection of cases for prediction analysis.

Out of the 912 patients, 639 patients were followed up for at least 5 years or until death from disease (event). Six out of the 12 variables had missing values ranging from 2–24% (M-stage 2%, N-stage 3%, T-stage 4%, type of surgery 5%, tumor size 10%, grade 24%). Since both artificial neural networks and LASSO require complete cases, all patients with missing values were excluded from the analysis. This resulted in a dataset consisting of 440 patients (Fig 1). Logistic regression was considered appropriate for comparison with the ANN due to the binary endpoint. Disease-specific survival (DSS) was used as endpoint (0 = alive, 1 = death from disease). DSS in the database is calculated from diagnosis to date of death, last date known to be alive, or until last follow-up. Patients who died from other causes were censored.

Variables examined included age at diagnosis (continuous), gender (male or female), tumor size (cm), tumor location (head versus other), surgery (partial or total pancreatectomy), histological grade (well differentiated, moderately differentiated and poorly differentiated/anaplastic), radiotherapy (yes or no), chemotherapy (yes or no/unknown if administered), year of diagnosis (2004–2016) and staging according to the American Joint Committee on Cancer (AJCC) separated on T, N and M stage. In the database the 6^th^ edition AJCC stage [13] was available for patients diagnosed between 2004 to 2009 and the 7^th^ edition AJCC stage [14] for patients diagnosed between 2010 to 2016. Both editions share the same definitions of T, N and M stages, thereby ensuring uniformity of staging during the study period. This added up to twelve variables: age, gender, year of diagnosis, tumor location, radiotherapy, chemotherapy, T-stage, N-stage, M-stage, surgery, tumor size, grade. Continuous data are presented as median with interquartile range and standard deviation. Categorical data are presented as frequencies with percentages (Tables 1 and 2). The type of surgery coded as “surgery NOS” (not otherwise specified) and “pancreatectomy NOS” were classified as missing due to uncertainty in coding of variable of interest (total or partial pancreatectomy). Chemotherapy is in SEER coded as administered (yes) or not administered (no) and unknown if administered (no). The sequence in respect to surgery is not stated. In the case of radiotherapy, >90% of those who received it had it following surgery, some prior to surgery and only a few both prior and following surgery.

**Table 1:** Performance measures used in assessing the chosen models.

	Definition
**Accuracy**	(TP+TN) / total
**Precision (PPV)**	TP / (TP+FP)
**Recall (sensitivity)**	TP / (TP+FN)
**F1 score**	2 * ((PPV * Recall) / (PPV + Recall))
**NPV**	TN / (TN+FN)
**Specificity**	TN / (TN+FP)

FN false negative. FP false positive. LR likelihood ratios. NPV negative predictive value. PPV positive predictive value. TN true negative. TP true positive.

**Table 2:** Variables of the 440 cases with complete data used in prediction analysis.

Variables	N (%) or median (IQR), SD
**Age at diagnosis**	66 (58–73), 11.3
**Year of diagnosis (2004–2016)**	41, 38, 52, 38, 48, 42, 46, 53, 27, 21, 14, 15, 5
**Tumor size (cm)**	3.5 (2.5–5), 2.9
**Gender (male vs female)**	235 vs 205 (54 vs 46)
**Site (pancreatic head vs other)**	291 vs 149 (66 vs 34)
**Type of surgery (partial vs total pancreatectomy)**	378 vs 62 (86 vs 14)
**Radiotherapy (no vs yes)**	304 vs 136 (69 vs 31)
**Chemotherapy (no/unknown vs yes)**	186 vs 254 (42 vs 58)
**T stage**	
** T1**	42 (9)
** T2**	82 (19)
** T3**	283 (64)
** T4**	33 (7)
**N stage (0 vs 1)**	203 vs 237 (46 vs 54)
**M stage (0 vs 1)**	406 vs 34 (92 vs 8)
**Histological grade**	
** Well differentiated**	96 (22)
** Moderately differentiated**	222 (50)
** Poorly differentiated/anaplastic**	122 (28)

IQR interquartile range. SD standard deviation.

### Statistical analysis and predictive models

Before any analyses were conducted, 30% of the total number of patients were randomly put aside to be used as a test set. These patients were not used in any modelling or analyses before being used to test the models. The remaining patients will be referred to as model data/set. To validate the comparability between the model and test set the Chi-squared test (or Fischer’s exact test if appropriate) was used for categorical variables and Mann-Whitney U test for continuous variables as well as the log-rank test for survival-analysis. A 2-sided p-value of less than 0.05 was considered statistically significant. These calculations were performed following modelling and testing of the predictive models and performed using SPSS version 25.0 (IBM, Armonk, New York, USA). All analyses on modelling were performed in R v. 3.6.3 [15–18].

**Fig 2:**
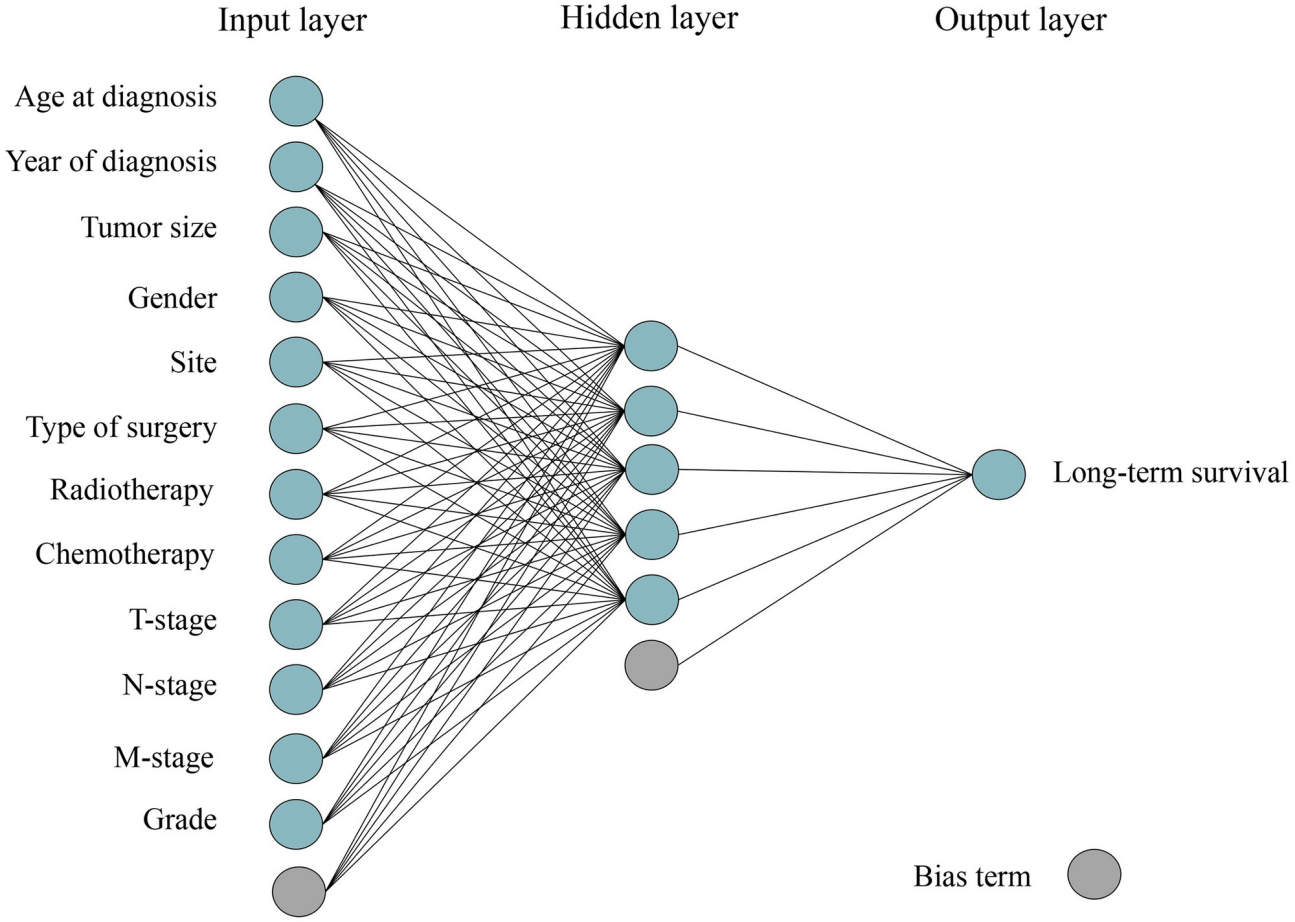
Schematic structure of the applied multilayer perceptron neural network with three layers.

A multilayer perceptron ANN was created consisting of three layers (input, hidden and output) (Fig 2). Two different ANN models were used, one using a reduced set of variables (ANN model 1) and one including all 12 variables (ANN model 2). The modelling data was split into training data and validation data (containing 50% and 20% of the patients from the dataset, respectively). This split was conducted eleven times, with models trained on each training set, and evaluated on each validation set. The final prediction of the ANN is a majority vote ensemble prediction, using all eleven models.

The process for selecting the best subset of variables starts with the ANN using all variables. The optimal number of hidden nodes, as well as the optimal weight decay is estimated using 10-fold cross validation. Next using the cross validated values, several different networks, each excluding one variable, are trained and evaluated. The average number of correctly classified observations across all eleven sets of data is then used to determine which variable contributes the least to the model. That variable is eliminated, then new values for the number of hidden nodes and weight decay are estimated. Networks with one less variable are fitted and evaluating using the same process. This continues until the network consists only of a single variable. The network which achieved the highest number of correctly classified observations in the validation data is chosen. Finally, new values for the number of hidden nodes and the weight decay is re-estimated. Following the above steps, the resulting ANN model 1 had 1 hidden node and a weight decay of 0.01. The included variables were age at diagnosis, T-stage 3 and 4 (T-stage 1 and 2 combined as reference category) and N-stage. The resulting ANN model 2 had 8 hidden nodes and a weight decay of 0.3.

A LASSO was conducted in order to select the best variables and a logistic regression using the variables selected in the LASSO was performed. The regularization parameter (lambda) was estimated using 10-fold cross validation. This resulted in a model with a lambda value of 0.023. The selected variables in the LASSO-model were age, year of diagnosis, gender, tumor size, tumor location, N-stage, M-stage, T-stage (2, 3 and 4 with T-stage 1 as reference category) and grade (moderately and poorly differentiated/anaplastic with well differentiated as reference category). A logistic regression using all variables was fitted, corresponding to the ANN with all variables included.

Once all models were calculated, their performance was evaluated based on their predictive performance on the test data. Performance was measured by calculating accuracy, precision, recall, and F1 score. F1 score can be interpreted as a balance between precision and recall (Table 1). A 95% confidence interval was calculated with Wilson score interval for all measures excluding the F1 score, which was performed by bootstrapping with 10 000 repetitions. McNemar´s test was performed to statistically compare the two models selecting parameters, i.e. ANN model 1 and LASSO and the two models including all parameters, i.e. ANN model 2 and logistic regression model. Venn diagrams on recall (sensitivity) were created to illustrate the models predictive ability for identification of the cases with disease-specific mortality in the test dataset.

## Results

### Patient demographics

The 440 cases with complete data were included in the prediction analysis (Table 2). The median age was 66 and the median tumor size was 3.5 cm. The distribution of male and female was almost 1:1. Most invasive IPMN engaged the head of the pancreas (66%) and a partial pancreatectomy was most often performed (86%). Chemotherapy was known administered in a majority of cases while a third received radiotherapy. About 1 in 4 received both radiotherapy and chemotherapy. Variables related to tumor characteristics showed a high percentage of higher T-stages, i.e. T-stage 3 and 4, lymph node metastasis in 54%, almost 1 in 10 with distant metastasis and about 3 in 4 having low tumor grade, i.e. well to moderate differentiation. A total of 353 (80%) died during the five-year period with 336 (76%) from the disease. The included 440 cases with complete data showed almost no difference in baseline data to the 639 cases they were derived from. The differences seen were a slightly higher percentage of patients receiving chemotherapy (52% and 58%), cases graded as T-stage 3 (56% and 64%) and cases graded as N-stage 1 (46% and 54%) in the complete cases dataset.

### Model and test set

Following modeling on 308 cases, test of the models on the randomly prior selected 132 cases were performed. Of the 132 cases, mortality from the disease was observed in 99 (75%) cases and 33 cases had survived five years or died of disease unrelated causes. Following testing, comparison between the model set and the test set was performed showing no difference in survival (log-rank test, p = 0.603). The only variable showing statistical difference between the two datasets was radiotherapy with a higher percentage of patients receiving radiotherapy in the test set (222 vs 86 and 82 vs 50, p = 0.038).

### Validation of variable selection models

The ANN model selecting a subset of variables (ANN model 1) showed an accuracy of 0.82 and an F1 score of 0.89 corresponding to a precision and recall of 0.83 and 0.95, respectively.

**Fig 3:**
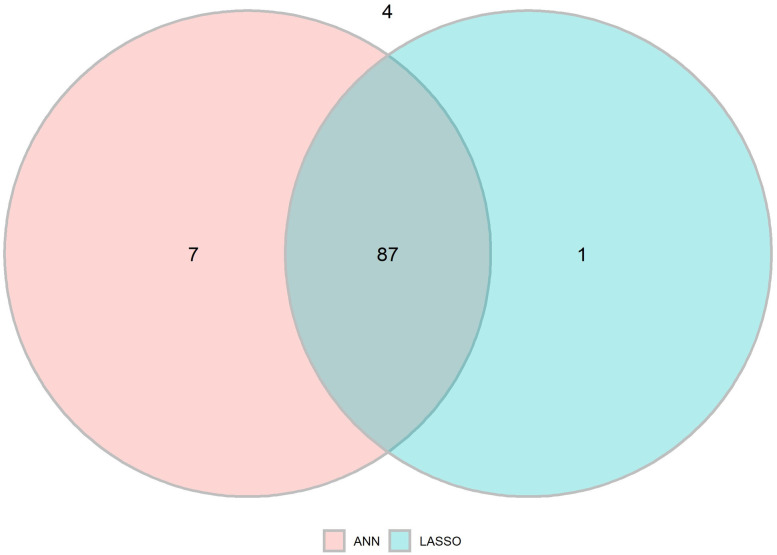
Venn diagram on recall (sensitivity) of ANN model 1 and LASSO regression model. Seven correctly classified by ANN and not LASSO, 1 by LASSO and not ANN, 87 by both models and 4 not correctly classified by any model.

The corresponding logistic regression model (LASSO-model) had an accuracy of 0.80 and F1, precision and recall of 0.87, 0.85 and 0.89 respectively (Table 3). McNemar´s test resulted in p = 0.627. The Venn diagram illustrates the predictive ability in classifying the true positive (DDS = 1) of ANN model 1 compared to the LASSO model, with the ANN model 1 correctly classifying 7 patients that the LASSO did not (Fig 3).

**Table 3:** Performance including 95% confidence interval of the four different models used in the prediction of 5-year disease-specific survival.

	ANN model 1	LASSO-model	ANN model 2	Logistic regression
**Accuracy**	0.82 (0.74–0.87)	0.80 (0.72–0.86)	0.81 (0.74–0.87)	0.81 (0.74–0.87)
**Precision**	0.83 (0.75–0.89)	0.85 (0.76–0.90)	0.85 (0.77–0.90)	0.86 (0.78–0.91)
**Recall**	0.95 (0.89–0.98)	0.89 (0.81–0.94)	0.91 (0.84–0.95)	0.90 (0.82–0.94)
**F1 score**	0.89 (0.84–0.93)	0.87 (0.81–0.91)	0.88 (0.83–0.92)	0.88 (0.83–0.92)
**NPV**	0.74 (0.51–0.88)	0.61 (0.42–0.76)	0.65 (0.46–0.81)	0.64 (0.46–0.79)
**Specificity**	0.42 (0.27–0.59)	0.52 (0.35–0.68)	0.52 (0.35–0.68)	0.55 (0.38–0.70)
**Hidden nodes**	1	NA	8	NA
**Weight decay**	0.01	NA	0.3	NA
**Lambda**	NA	0.023	NA	NA

ANN artificial neural network. LASSO least absolute shrinkage and selection operator. NA not applicable. NPV negative predictive value. ANN model 1: Variable selection process was carried out. ANN model 2: All twelve variables were included in the network.

### Validation of complete variable models

**Fig 4:**
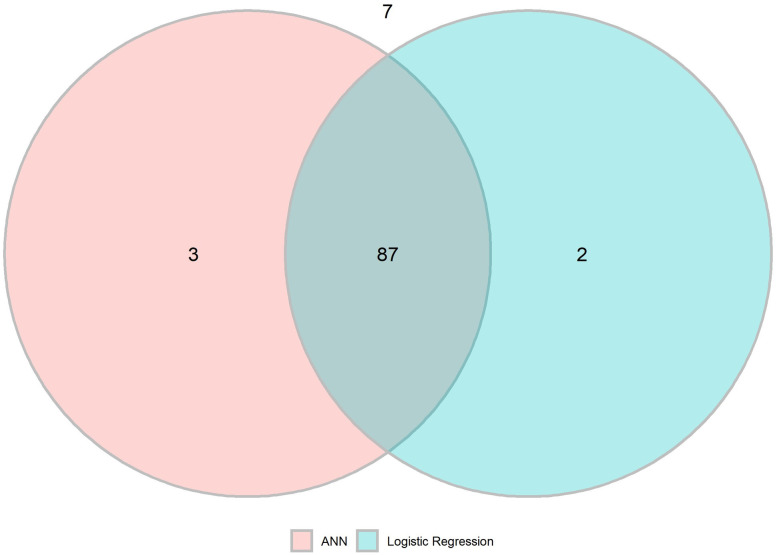
Venn diagram on recall (sensitivity) of ANN model 2 and logistic regression model. Three correctly classified by ANN and not LASSO, 2 by LASSO and not ANN, 87 by both models and 7 not correctly classified by any model.

The ANN model using all variables (ANN model 2) performed similar to the logistic regression (LR) with the same accuracy and F1 score of 0.81 and 0.88, respectively. The precision and recall were 0.85 versus 0.86 and 0.91 versus 0.90 for the ANN and LR, respectively (Table 3). McNemar´s test resulted in p = 1.000. The similar results in the aspect of recall between the models are illustrated in Fig 4.

## Discussion

This is to our knowledge the first study investigating the performance of ANN and LASSO for predicting long-term survival in surgically resected invasive IPMN. ANN have previously been used in survival analyses of PDAC [8]. For IPMN, ANN have been used in radiomics for classifying pancreatic cystic neoplasms [19]. Deep learning algorithms have also been developed for classification of high-risk IPMN on MRI [20] and endoscopic ultrasonography (EUS) [21], as well as for differentiating between malignant and benign pancreatic cysts [22]. LASSO have been used in optimizing a PCR-based assay on cyst fluid predicting IPMN with high malignant potential [23]. Variable selection for ANN with LASSO has been proposed [24].

In this study, ANN models were developed and compared to regression models. One ANN using a variable selection process (ANN model 1) and one using all included variables (ANN model 2). The corresponding regression models were LASSO (variable selection method) and logistic regression including all variables.

All models performed fairly accurate predictions (approximately 0.80) in predicting survival status five year following diagnosis and received treatment (surgery and in a majority of cases chemotherapy and/or radiotherapy). McNemar´s test did show no statistical differences between the models. The ANN model 1 did, however, with only 3 variables, i.e. age, T-stage and N-stage perform especially good on recall (sensitivity) and negative predictive value (NPV) with 0.95 and 0.74, respectively. Recall and NPV corresponds, in our models, to a good performance in finding cases succumbing to the disease before five years. Selection and reduction of variables is important to reduce complexity and may, as shown by the performance of the ANN model 1 and to some degree the LASSO-model, not negatively impact model performance.

The outcome chosen, 5-year disease-specific survival, is rarely described in research on pancreatic cancer [25]. However, 5-year survival is considered a benchmark in cancer prognosis and is thus an important outcome measure, especially following resection of curable attempt.

Of the variables used in this study, the different components of the AJCC stage were analyzed separately and the AJCC stage was not added due to their multicollinearity. Tumor size was used as T-stage involves the relation of tumor growth to the surrounding tissue. The year of diagnosis was included to represent treatment development during the study period (2004–2016).

Several limitations need to be addressed. This is a retrospective study using data that relies on the inputs given in the database. It contains patients from different institutions and during a wide time period with possible different management strategies. However, large cancer registries offer a great source of information in rare diseases such as invasive IPMN [13]. There were no data on the different subtypes of invasive IPMN (tubular or colloid), which may have different outcomes [4,5] and entail different management strategies. However, according to recent guidelines management of resected invasive IPMN do not differ in regard to subtype [3]. There is uncertainty regarding neoadjuvant and adjuvant therapy and also lack of information of reasons for the choice of administration (including completeness of planned therapy) with possible selection bias. Both chemotherapy and radiotherapy variables should thus be viewed with caution [14]. There was a high percentage of missing values (4% for whole dataset), which somewhat limited the number of eligible cases for prediction analysis. However, there was no substantial difference between the 440 cases with complete data compared to the 639 cases. Imputation of missing values was considered; however, the added value was deemed limited. All four models had a relatively low specificity, corresponding to misclassification of true negatives (survivors). However, this must be put into perspective with the models ability to correctly classify those with high risk of not surviving. The binary outcome was somewhat imbalanced to a 3:1 ratio (diseased:survivors), both in the complete data set as well as in the test set. This was, considering the size of the datasets, deemed not to influence the modelling or prediction outcome in any major way. For the same reason, the simpler standard cross validation and not a stratified one was performed.

## Conclusions

The present study shows the feasibility and validity of ANN as well as LASSO for survival prediction in invasive IPMN. Twelve general and easily assessable clinico-pathological variables were used to build the prediction models. The performances of the ANN and LASSO models were comparable to traditional logistic regression. Thus, we could not observe a major performance improvement using these more sophisticated statistical methods. As knowledge of IPMN increases at the molecular level, it may be possible to add genomic and proteomic data to these models to improve outcome prediction.

## Supporting information

S1 Data (XLSX)
